# In silico analysis suggests disruption of interactions between *HAMP* from hepatocytes and *SLC40A1* from macrophages in hepatocellular carcinoma

**DOI:** 10.1186/s12920-021-00977-0

**Published:** 2021-05-17

**Authors:** Liang Hu, Chao Wu

**Affiliations:** 1 Department of Thyroid Surgery, The First Affiliated Hospital, Zhejiang University School of Medicine, Hangzhou, China; 2 State Key Laboratory for Diagnosis and Treatment of Infectious Diseases, National Clinical Research Center for Infectious Diseases, Collaborative Innovation Center for Diagnosis and Treatment of Infectious Diseases, The First Affiliated Hospital, Zhejiang University School of Medicine, Hangzhou, China

**Keywords:** Single-cell RNA sequencing, Cell-type-specific genes, Proliferative hepatocyte, *HAMP*-*SLC40A1* signaling

## Abstract

**Background:**

Identification of factors associated with proliferation in the hepatocellular carcinoma (HCC) microenvironment aids in understanding the mechanisms of disease progression and provides druggable targets. Gene expression profiles of individual cells in HCC and para-carcinoma tissues can be effectively obtained using the single-cell RNA sequencing (scRNA-Seq) technique. Here, we aimed to identify proliferative hepatocytes from HCC and para-carcinoma tissues, detect differentially expressed genes between the two types of proliferative hepatocytes, and investigate their potential roles in aberrant proliferation.

**Results:**

Two respective gene signatures for proliferative cells and hepatocytes were established and used to identify proliferative hepatocytes from HCC and para-carcinoma tissues based on scRNA-Seq data. Gene expression profiles between the two types of proliferative hepatocytes were compared. Overall, 40 genes were upregulated in proliferative hepatocytes from para-carcinoma tissue, whereas no upregulated genes were detected in those from HCC tissue. Twelve of the genes, including *HAMP*, were specifically expressed in the liver tissue. Based on previous reports, we found that *HAMP* modulates cell proliferation through interaction with its receptor *SLC40A1*. Comprehensive analysis of cells in HCC and para-carcinoma tissues revealed that: (1) *HAMP* is specifically expressed in hepatocytes and significantly downregulated in malignant hepatocytes; (2) a subset of macrophages expressing *SLC40A1* and genes reacting to various infections is present in para-carcinoma but not in HCC tissue. We independently validated the findings with scRNA-Seq and large-scale tissue bulk RNA-Seq/microarray analyses.

**Conclusion:**

*HAMP* was significantly downregulated in malignant hepatocytes. In addition, a subset of macrophages expressing *SLC40A1* and genes reacting to various infections was absent in HCC tissue. These findings support the involvement of *HAMP*-*SLC40A1* signaling in aberrant hepatocyte proliferation in the HCC microenvironment. The collective data from our in silico analysis provide novel insights into the mechanisms underlying HCC progression and require further validation with wet laboratory experiments.

**Supplementary Information:**

The online version contains supplementary material available at 10.1186/s12920-021-00977-0.

## Background

Maintenance of proliferative signaling is a hallmark of cancer [1]. Healthy tissues carefully control their cell growth and division cycle and ensure cell number homeostasis, which preserves tissue architecture and function. Cancer cells emit sustained proliferative signals that activate progression of the cell cycle as well as support the formation and growth of tumor tissue. Although the liver is susceptible to cancer invasion, it is also an organ with the capacity to regenerate after surgical removal or chemical injury [2]. The regenerative process of a normal, healthy liver is predominantly dependent on hepatocyte proliferation, growth, and programmed cell death [3]. The ability to distinguish proliferative hepatocytes from hepatocellular carcinoma (HCC) and normal liver tissues and comparison of their gene expression profiles will aid us in understanding the mechanisms underlying aberrant proliferative signaling in malignant cells.

Single-cell RNA sequencing (scRNA-Seq) is a powerful tool for profiling gene expression patterns in individual cells [4]. This technique provides an unprecedented opportunity to identify cells in vivo and comprehensively characterize their transcriptomes. Large-scale sequencing of single cells from multiple tissues of human and animal models, such as the Tabula Muris and the Mouse Cell Atlas (MCA) projects, facilitates the characterization of cells within their respective tissues, leading to enhanced understanding of the transcriptomes of individual cell types, especially those that are currently poorly characterized [5–7].

Normal and tumor human liver tissues have been examined using scRNA-Seq since 2018 [8]. Recently, Lu et al. released their scRNA-Seq data from HCC and para-carcinoma tissues (GSE149614). The non-tumor tissues include the para-carcinoma tissue, which is generally within 3 cm of the cancer foci's edge and the normal tissue that is at least 5 cm away from the cancer foci's edge. These primary datasets are used to identify and compare proliferative hepatocytes between non-tumor and HCC tissues.

*HAMP* is a crucial regulator of iron entry into the circulation in mammals [9]. In tumor cells, pathways of iron acquisition, efflux, storage, and regulation are disrupted, suggesting that the reprogramming of iron metabolism is a central aspect of tumor cell survival [10]. Previously, Vela and Vela-Gaxha [11] analyzed *HAMP* expression in HCC. Their results suggested that by lowering liver *HAMP* levels, HCC cells could secure abundant iron from sources such as enterocytes and macrophages. Shen et al. [12] reported that low *HAMP* expression is linked with higher rates of metastasis and poor disease-free status in HCC, and that the role of *HAMP* in cellular proliferation and metastasis is related to cell cycle checkpoints. The group proposed that *HAMP* serves as a tumor suppressor gene.

*HAMP* is believed to be involved in host defense, and when induced, it depletes extracellular iron to prevent its use by invading pathogens [13]. However, the issue of whether *HAMP* exerts additional effects on host defense mechanisms remains unclear. Ramakrishnan et al. [14] reported that *HAMP*-*SLC40A1* signaling modulates the proliferation of human pulmonary artery smooth muscle cells (hPAMSC). Under conditions of increased expression, *HAMP* binds *SLC40A1* to form a complex that undergoes internalization and degradation, leading to further enhancement of iron retention in cells. Simultaneously, higher proliferation of hPASMCs was observed, suggesting that iron retention encourages a proliferative state.

Based on the scRNA-Seq data from the Tabula Muris project and RNA-Seq data from the Genotype-Tissue Expression (GTEx) project, we identified a cell-type-specific gene signature for hepatocytes. In a previous study, our group determined a cell-type-specific gene signature for proliferative cells. Here, we identified proliferative hepatocytes in HCC and para-carcinoma tissues using the two above gene signatures with scRNA-Seq data and evaluated the gene expression profiles of the two types of proliferative hepatocytes. We found that *HAMP* was specifically expressed in hepatocytes and significantly downregulated in malignant hepatocytes. We also revealed that a subset of macrophages expressing *SLC40A1* and genes reacting to various infections was present in para-carcinoma but not in HCC tissue. These findings were independently validated with scRNA-Seq and large-scale tissue bulk RNA-Seq/microarray analyses. The data obtained using our in silico approach strongly suggest the role of *HAMP*-*SLC40A1* signaling in aberrant hepatocyte proliferation in the HCC microenvironment.

## Methods

### Data sets

We downloaded the scRNA-Seq dataset of cells from 81 cell types from the Tabula Muris project. ScRNA-Seq datasets of cells from HCC and para-carcinoma tissues (GSE149614), normal liver tissue (GSE115469), and HCC tissue (GSE125449) from the GEO database were obtained. The RNA-Seq dataset for normal tissue samples of 54 tissue types from the GTEx project was downloaded. We also downloaded the RNA-Seq dataset of developing mouse liver samples from the GEO database (GSE132034) and extracted the RNA-Seq dataset for tumor and para-carcinoma tissue samples of 32 tumor types, including HCC, from the TCGA project. Clinical information regarding the HCC samples was acquired, along with a microarray dataset for HCC and para-carcinoma tissues from the GEO database (GSE36376). Details are listed in Additional file 1: Table S11.

### Gene expression data preprocessing

Acquisition and normalization of the expression of each gene from individual datasets are described below.

1. For the scRNA-Seq dataset from the Tabula Muris project, we grouped all single cells into 81 types according to annotation and counted the number of cells belonging to each cell type. For gene and cell types, we counted the number of cells expressing the gene within the cell type, which was calculated as a percentage. The percentage value was selected as the normalized gene expression level for that cell type. Normalized expression levels for all *Mus musculus* genes in the 81 cell types were obtained using this technique.
2. For scRNA-Seq datasets of GSE149614 and GSE125449, we first downloaded the read count matrix. GSE125449 had two read count matrices, which were merged. Next, we recorded the reads for each gene in each cell in the datasets. For cell $i$, the read counts of all the genes were summed to obtain the read depth $x$. For gene $j$ with a read count of $y$, we calculated expression levels (RP10K: Reads Per 10 Kilo) in cell $i$ as $\frac{y}{x}*10000$. Expression levels for all genes in cells were obtained using this technique.
3. For the scRNA-Seq dataset GSE115469, log2CPM (counts per million) gene expression values were directly downloaded.
4. For the tissue RNA-Seq dataset from the GTEx project, TPM (transcripts per million) profiles for all genes were downloaded.
5. For the mouse liver development RNA-Seq dataset GSE132034, FPKM (fragments per kilobase of transcript per million mapped reads) gene expression values were directly downloaded.
6. For the tumor and para-carcinoma tissue RNA-Seq dataset from the TCGA project, the RSEM method was used to process gene-level normalized counts for all genes, which were used as gene expression levels.
7. For the HCC tissue microarray dataset GSE36376, the quartile normalized gene expression values were directly downloaded.

For each dataset listed in 2 to 7, expression levels of a single gene across all samples were determined and normalized via the Z-score transformation. We normalized the expression levels of all genes in a dataset using this method.

### Expression heatmap and hierarchical clustering analysis

Hierarchical clustering analysis was conducted to group genes based on normalized expression levels within different types of RNA-Seq data. We employed the R package "factoextra" for clustering analyses. The "Euclidean" method was employed to measure the distance between the observations, "ward.D2" was selected for agglomeration of the observations, and the "fviz_dend" function was used to visualize the dendrogram. Expression heatmaps were generated, and the hierarchical clustering analyses conducted are described below.

1. The scRNA-Seq dataset from the Tabula Muris project

**Fig. 1:**
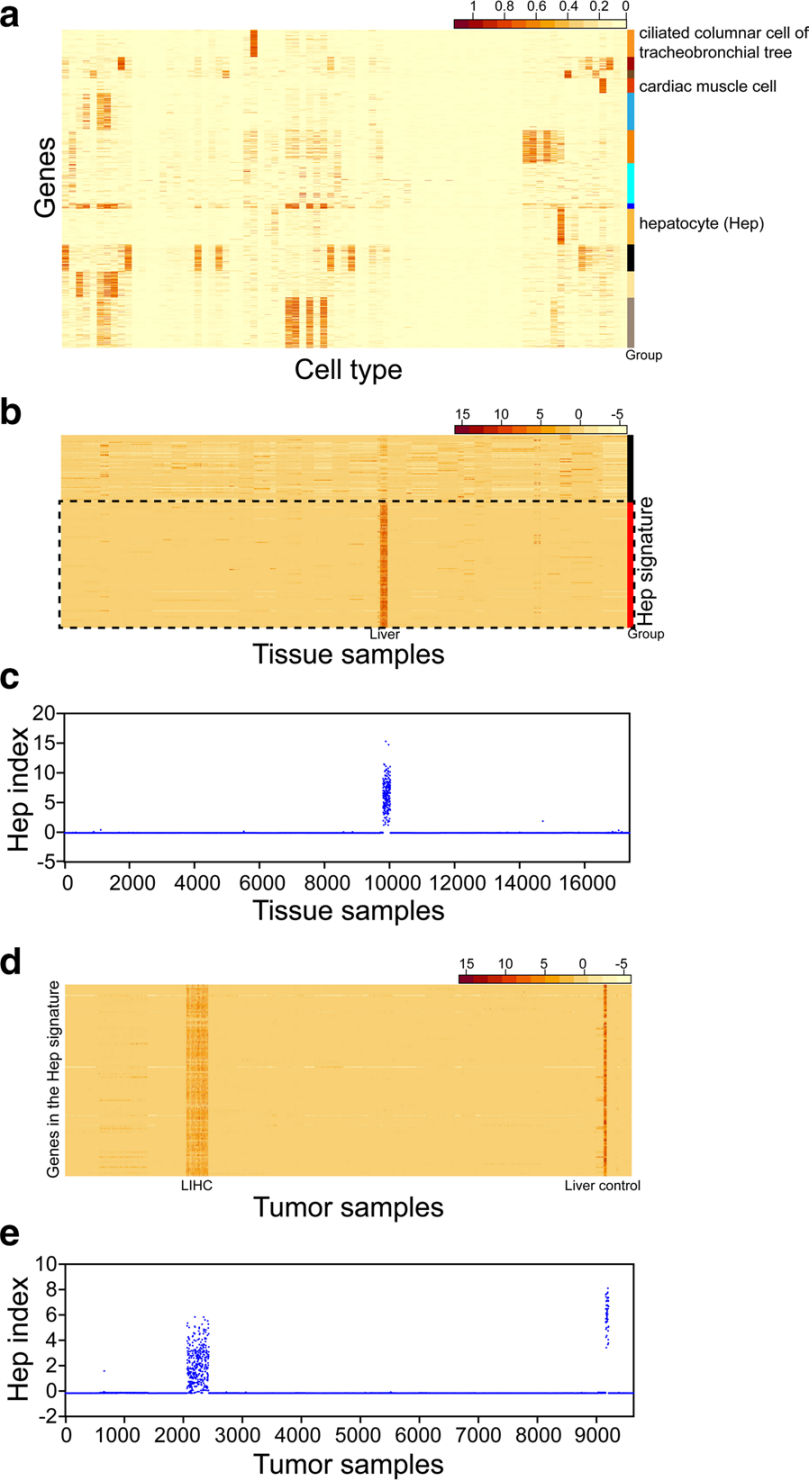
A set of 142 genes specifically expressed in human hepatocytes. **a** Gene expression profiles of 81 cell types. Columns present the gene expression profiles of specific cell types. The listed cell types match the 81 types in Additional file 2: Table S1 in sequence. Hierarchical clustering according to gene expression was conducted across the 81 cell types. The "group" marker represents their classifications. **b** Expression profiles of the 225 genes, showing a specific transcription pattern in mouse hepatocytes in samples across 54 human tissues from the GTEx project. Each column presents the gene expression profile of a sample. Hierarchical clustering according to gene expression was conducted. The "group" marker represents their classifications. **c** Expression of the Hep gene signature (Hep index) of samples across the 54 human tissues. **d** Expression profiles of the 142 genes belonging to the Hep gene signature in samples across 32 tumor types from the TCGA project. Each column presents the gene expression profile of a sample. **e** Hep index of samples across 32 tumor types from the TCGA project

In Fig. 1a, normalized expression levels of all *Mus musculus* genes in the 81 cell types were obtained from the Tabula Muris project. We subsequently identified genes with normalized expression levels less than a threshold of 0.1 in eight cell types, specifically, (1) stem cells of the epidermis, (2) Slamf1-positive multipotent progenitor cells, (3) megakaryocyte-erythroid progenitor cells, (4) late pro-B cells, (5) granulocyte monocyte progenitor cells, (6) granulocytopoietic cells, (7) common lymphoid progenitors, and (8) pre-natural killer cells. Genes with normalized expression levels greater than 0.5 were further selected from the other 73 cell types, leading to a total of 1817 genes. Next, genes were clustered by their normalized expression levels across the 81 cell types, and the clustering tree was subdivided into twelve groups. Genes were finally sorted according to clustering results, and a heatmap was generated based on normalized gene expression levels.

2. The tissue RNA-Seq dataset from the GTEx project

Normalized expression levels of 225 genes in all 17,382 tissue samples were initially obtained (Fig. 1b). Genes were clustered by their normalized expression profiles, and the clustering tree was sectioned into two groups. Finally, genes were sorted according to clustering results, and a heatmap was generated based on normalized expression levels.

3. The RNA-Seq dataset of tumor and para-carcinoma tissues from the TCGA project

**Fig. 2:**
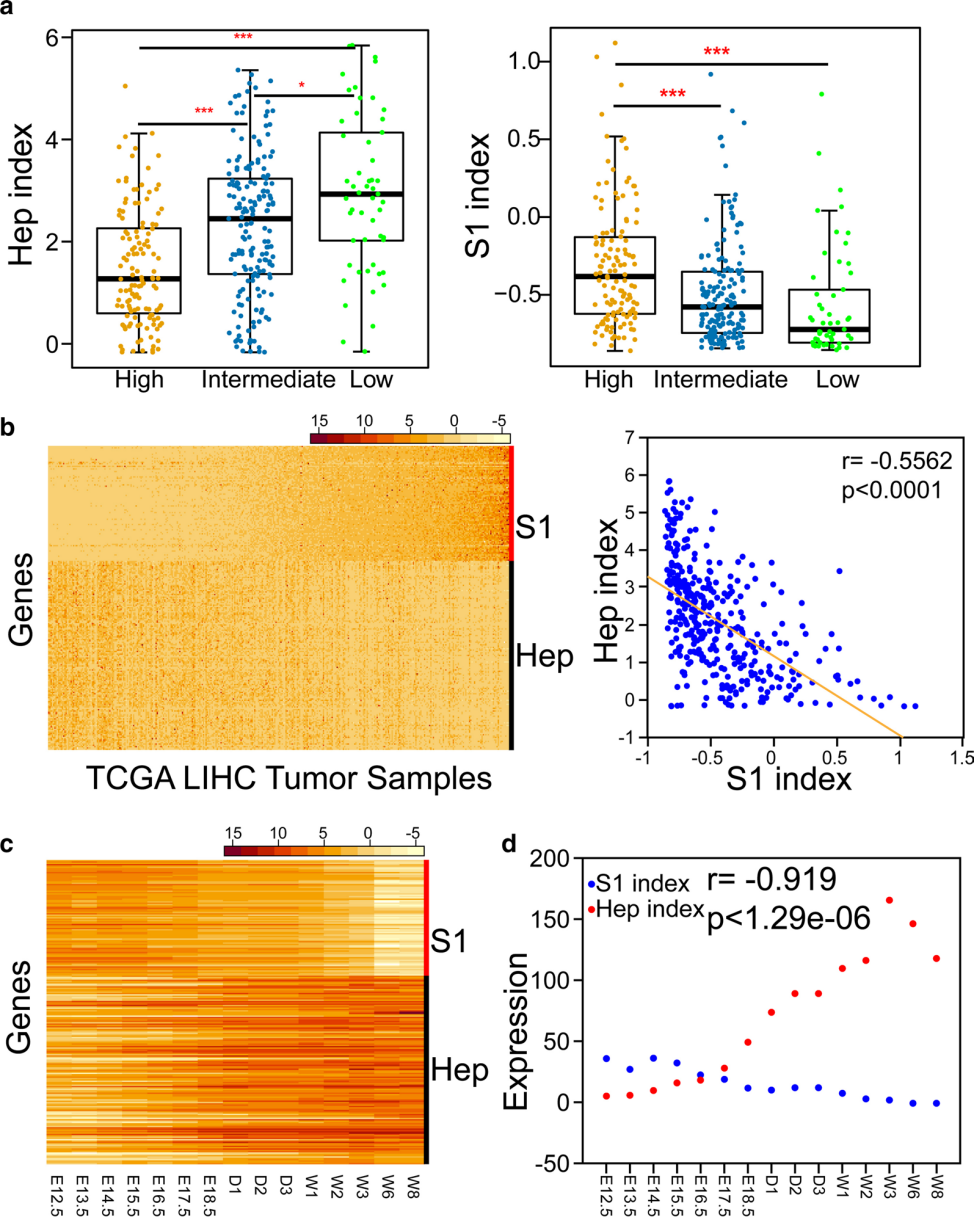
The Hep and S1 indexes are negatively correlated in tissues from HCC or developing liver. **a** Hep and S1 indexes from different histologically graded groups of HCC patients. The *t*-test was applied to compare the Hep and S1 indexes of patients from different groups. *** Signifies *P* values < 0.001 and * signifies *P* values < 0.05. **b** Expression profiles of genes from the S1 and Hep gene signatures and scatter plot of S1 and Hep indexes for HCC tissues. The Pearson correlation coefficient ($r$) between the two indexes was calculated, and the *P* value of $r$ was estimated. **c** Expression profiles of genes from the S1 and Hep gene signatures in liver tissues from different development stages. E, D and W denote embryonic day, day and week, respectively. **d** Plot of S1 and Hep indexes for liver tissues from different development stages. The Pearson correlation coefficient ($r$) between the two indexes was calculated, and the *P* value of $r$ was estimated

We initially obtained normalized expression levels of the 142 genes comprising the Hep gene signature in all 9630 tumor/para-carcinoma tissue samples (Fig. 1d), which were used to subsequently generate a heatmap. A heatmap was constructed with normalized expression levels of genes from the Hep and S1 gene signatures (Fig. 2b).

4. The mouse liver development RNA-Seq dataset GSE132034

We initially obtained normalized expression levels of the 142 genes of the Hep signature and the 87 genes from the S1 signature in 15 samples (Fig. 2c), which were used to generate a heatmap.

### Gene set expression analysis

We identified two gene signatures, S1 and Hep. The methods used to calculate the expression levels of the gene sets are described below.

1. Bulk RNA-Seq datasets from the GTEx and TCGA projects and mouse liver development.

We assessed expression of the Hep gene signature (Hep index) in the datasets, as shown in Figs. 1c, e, 2a, d, f. For a gene $i{ (}1 \le i \le 142{)}$ in the Hep gene signature and sample $k$ ($1 \le k \le N$) in a dataset with sample number $N$, normalized expression was obtained as $Z_{ik}$. For the sample $k$, the Hep index ($Hep_{k}$) was calculated as follows:

$$Hep_{k} = median\left( {Z_{1k} ,Z_{2k} , \ldots , Z_{142k} } \right).$$

Using this technique, the Hep index of each sample was calculated.

Expression of the S1 gene signature (S1 index) of each sample was calculated in a similar manner (Fig. 2a, b, d).

2. ScRNA-Seq data for GSE149614, GSE115469, and GSE125449.

**Fig. 3:**
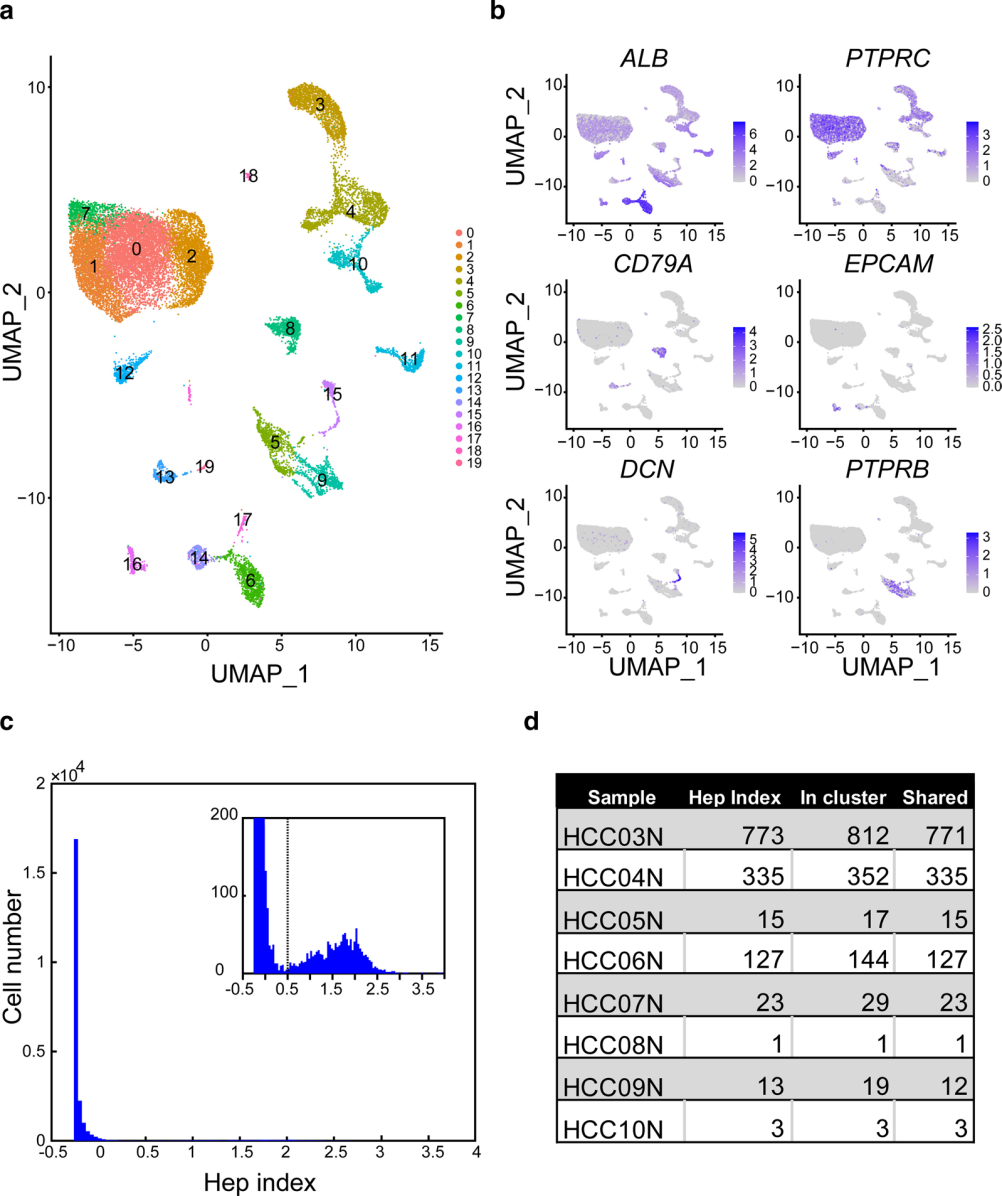
Identification of hepatocytes using the Hep index. **a** UMAP plot of cells from para-carcinoma tissue. **b** Heatmap of expression of known cell-type markers in the UMAP plot. **c** Distribution of the Hep index of all cells from para-carcinoma tissue. **d** Comparison of hepatocytes identified using cell-type markers and the Hep index. We counted cells with Hep index above 0.5, which were those in clusters 6, 14, and 17 of the UMAP plot, and shared cells between them in each para-carcinoma tissue sample. HCC03 to HCC10 represent patient codes. N indicates that three samples are from para-carcinoma tissue

**Fig. 4:**
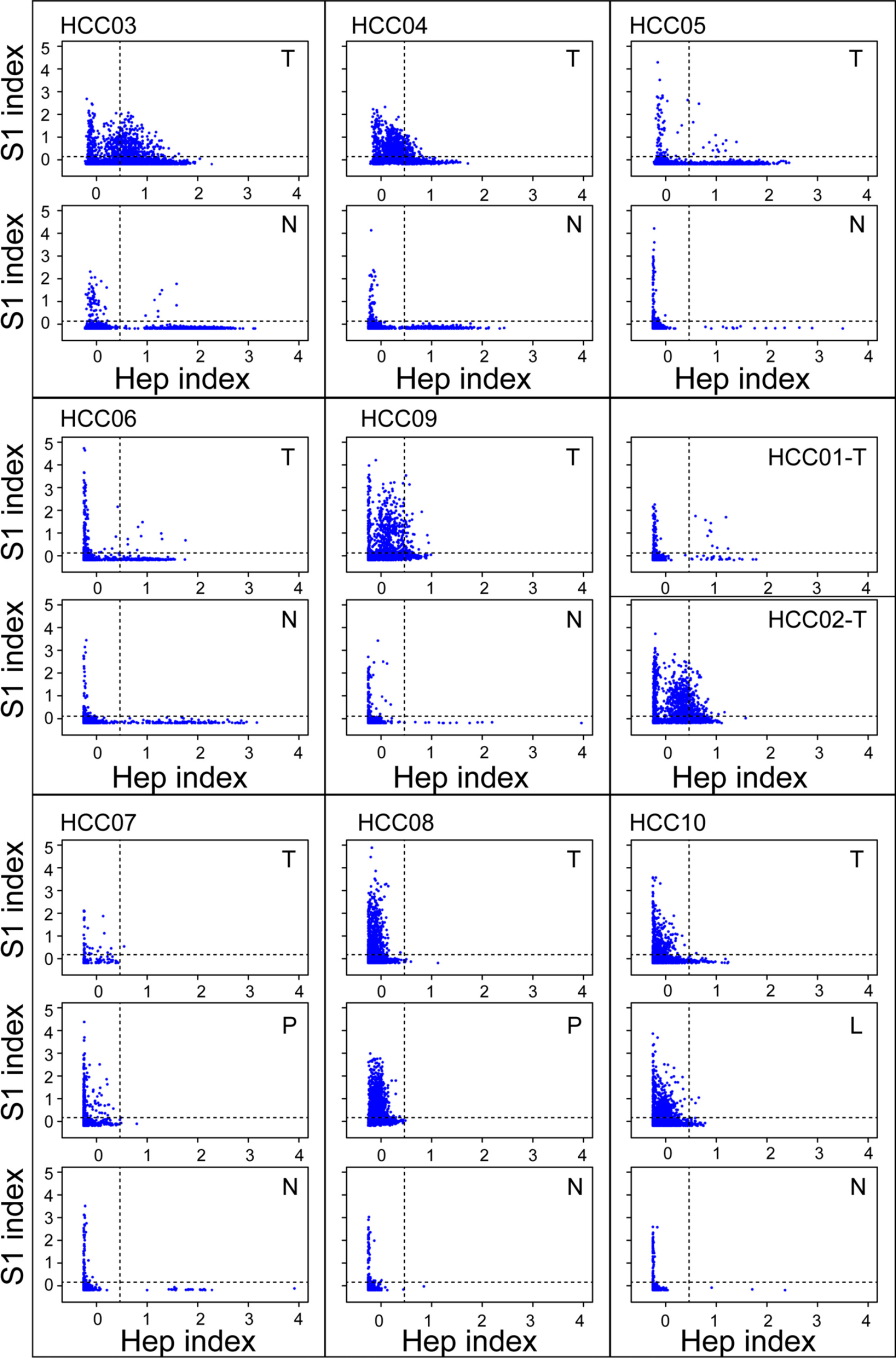
Scatter plots of S1 and Hep indexes for cells from HCC and para-carcinoma tissue samples of a patient. Each dot within the plot represents a cell. HCC01 to HCC10 represent patient sample codes. T, N, P, and L denote samples from tumor, para-carcinoma, portal vein tumor thrombus, and metastatic lymph node tissue samples, respectively. Thresholds of the Hep index (0.5) and S1 index (0.1) are additionally indicated in each plot

The Hep index in the datasets was calculated (Figs. 3c, 4, Additional file 5: S3a–d). For a gene $j{ (}1 \le j \le 142{)}$ in the Hep gene signature and cell $l$ ($1 \le l \le M$) in a dataset with the cell number of $M$, normalized expression was measured as $Z_{jl}$. For the cell $l$, the Hep index ($Hep_{l}$) was calculated as follows:

$$Hep_{l} = mean\left( {Z_{1l} ,Z_{2l} , \ldots , Z_{142l} } \right).$$

Using this method, the Hep index of each cell was calculated. The Hep index was assessed as the mean and not median value of normalized expression levels of the 142 genes, because the sequencing depth of scRNA-Seq is low, and a Hep index value of zero would be obtained for most cells if the median value is used as the Hep index. The S1 index of each cell was similarly calculated (Additional file 3: Figure S1, Fig. 4, Additional file 5: Figure S3c, d).

### Single-cell clustering

**Fig. 5:**
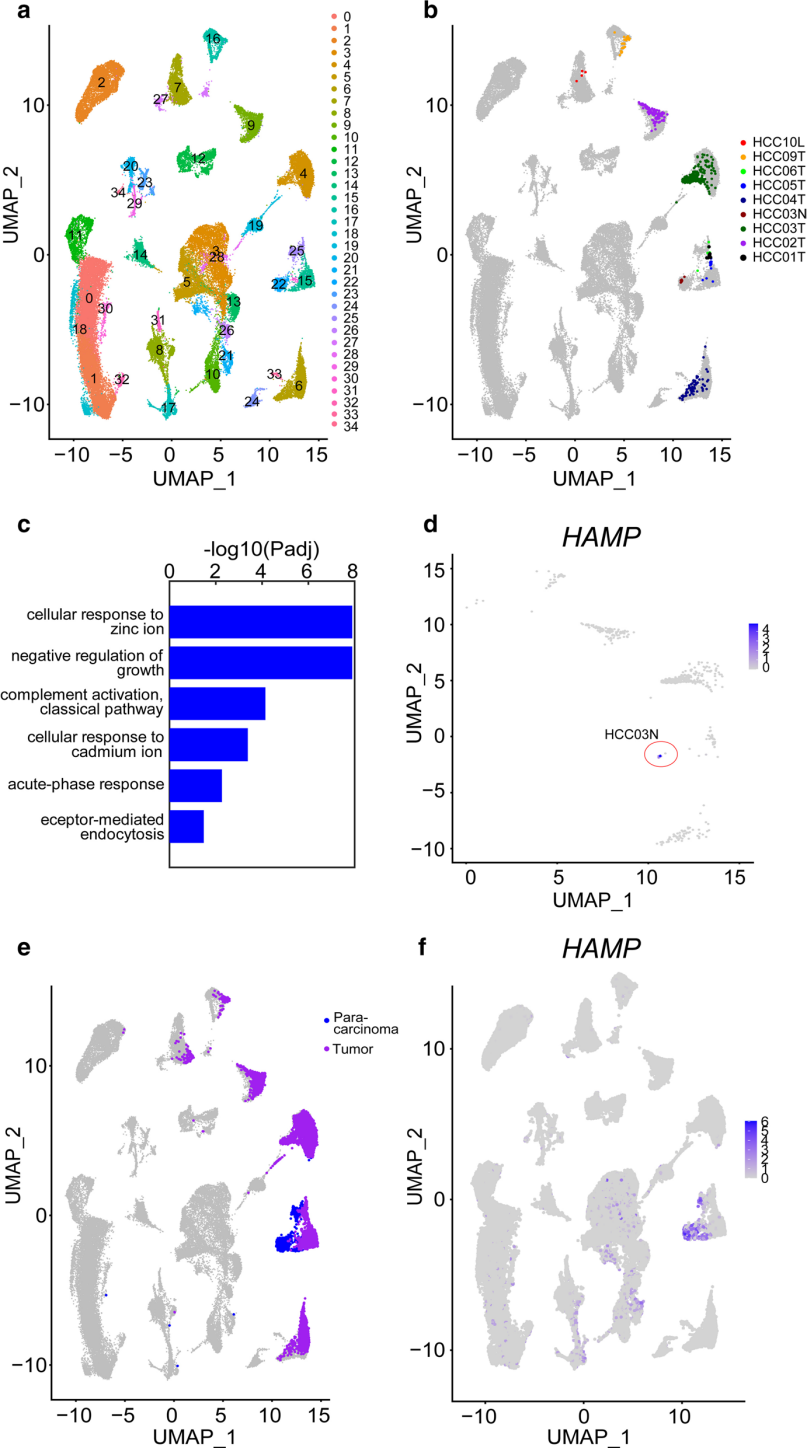
Proliferative hepatocytes in HCC and para-carcinoma tissues. **a** UMAP plot of all cells from HCC and para-carcinoma tissues. **b** Heatmap of proliferative hepatocytes from different samples in the UMAP plot. HCC01 to HCC10 represent patient sample codes. T and N signify samples from HCC and para-carcinoma tissue, respectively. **c** GO term enrichment of genes upregulated in proliferative hepatocytes from para-carcinoma tissue. **d** Heatmap of *HAMP* expression in proliferative hepatocytes from HCC and para-carcinoma tissue (HCC03N). **e** Heatmap showing hepatocytes (normal or malignant) from HCC and para-carcinoma tissue in the UMAP plot. **f** Heatmap showing *HAMP* expression in the UMAP plot

**Fig. 6:**
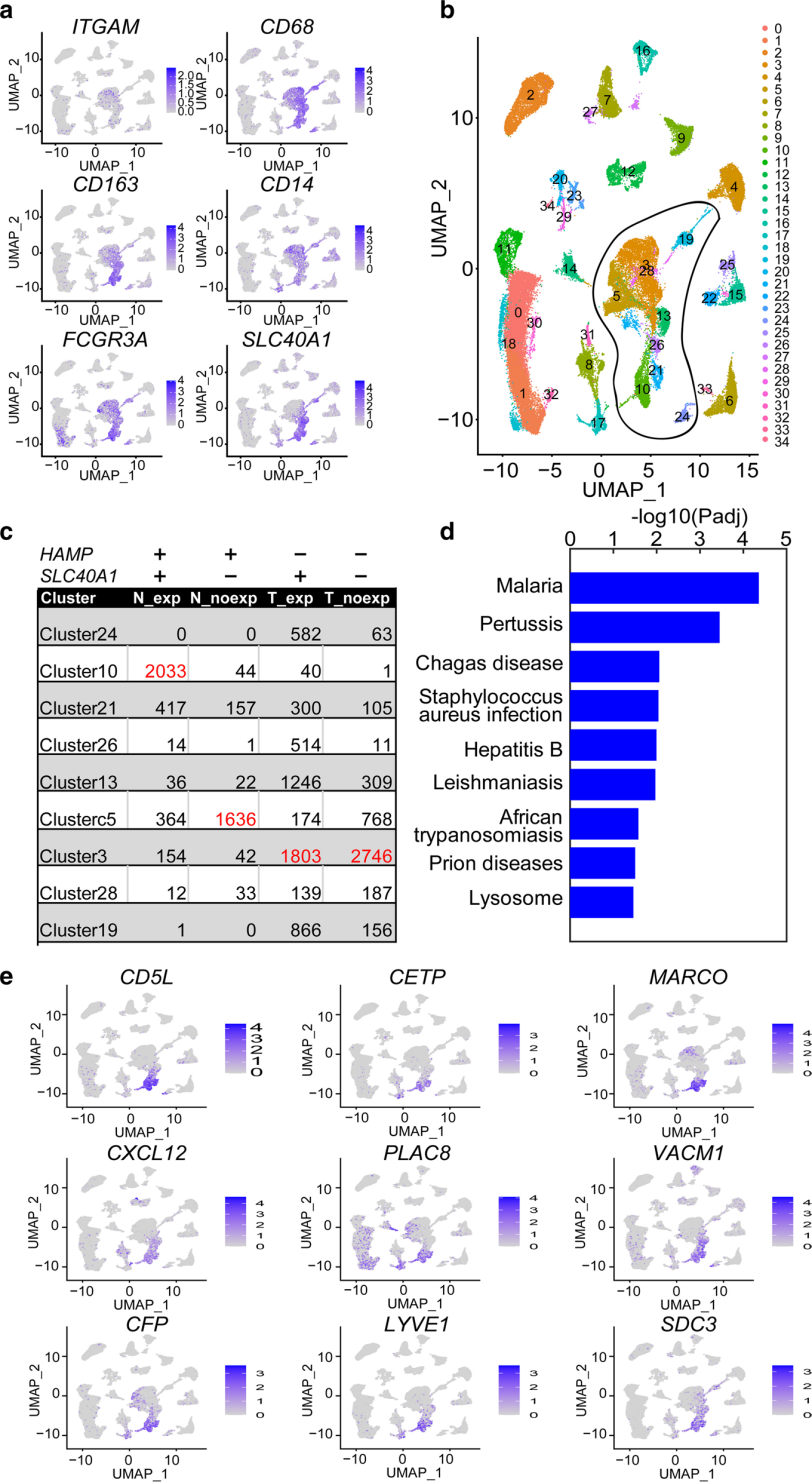
Macrophages in HCC and para-carcinoma tissue samples. **a** Heatmap of expression of known macrophage markers (*ITGAM*, *CD68*, *CD163*, *CD14*, and *FCGR3A*) and *SLC40A1* in the UMAP plot. **b** UMAP plot of all cells from HCC and para-carcinoma tissues. Clusters of macrophages are indicated. **c** Macrophages in the UMAP plot from different groups were counted. The N_exp group includes cells from para-carcinoma tissue expressing *SLC40A1*, and the N_noexp group includes cells from para-carcinoma tissue with no *SLC40A1* expression. The T_exp group includes cells from HCC tissues expressing *SLC40A1*, and the T_noexp group includes cells from HCC tissues with no *SLC40A1* expression. **d** KEGG pathway enrichment of marker genes of cluster 10 in the UMAP plot. **e** Heatmap showing expression of *CD5L*, *CETP*, *MARCO*, *CXCL12*, *PLAC8*, *VACM1*, *CFP*, *LYVE1*, and *SDC3* in the UMAP plot

**Fig. 7:**
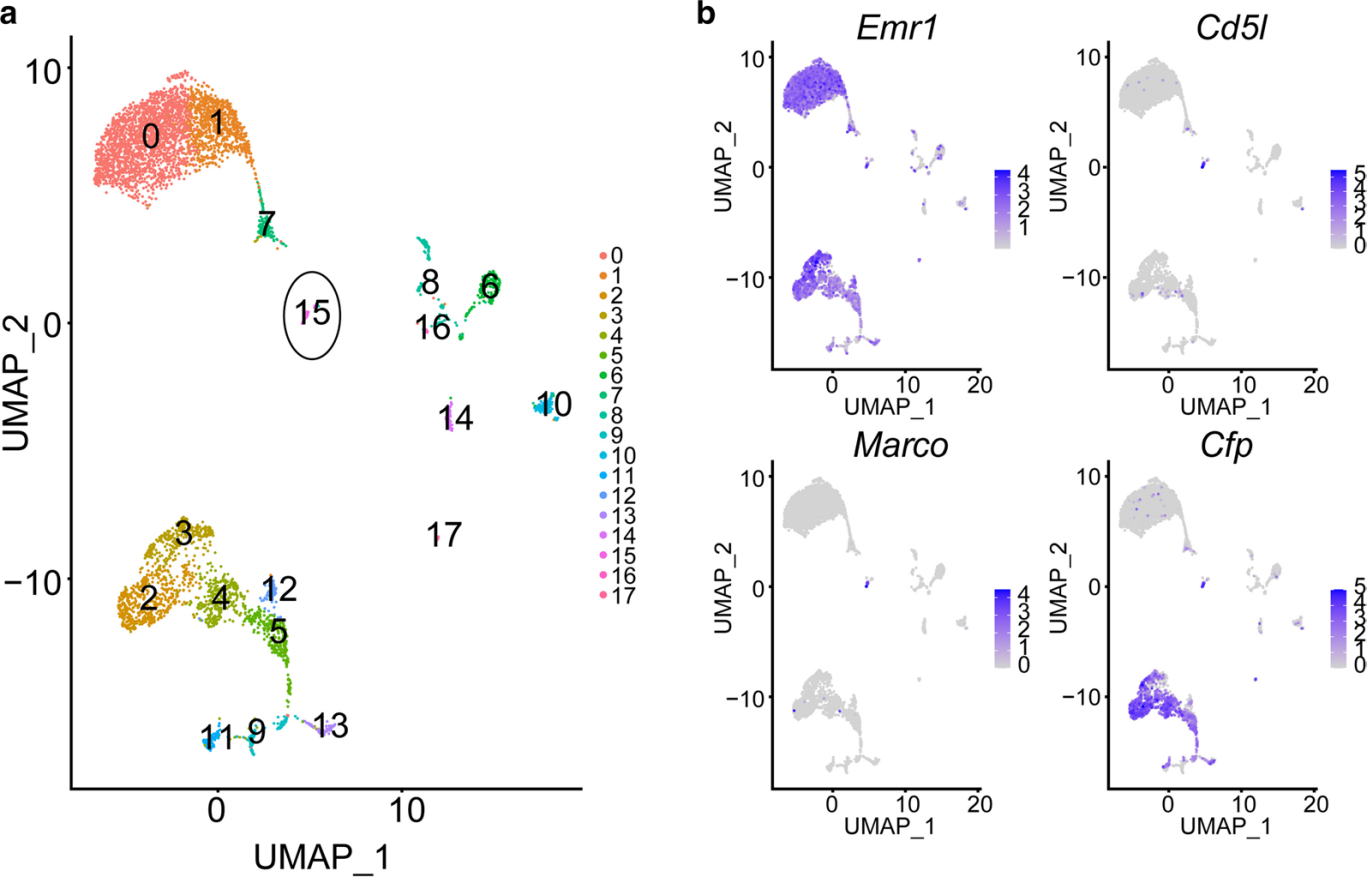
Expression of *Cd51*, *Marco*, and *Cfp* in macrophages from mouse organs and tissues. **a** UMAP plot of cells expressing *Emr1* in 20 mouse organs and tissues from the Tabula Muris project. **b** Heatmap showing expression of the mouse macrophage markers *Emr1*, *Cd51*, *Marco*, and *Cfp* in the UMAP plot

Cell clustering was performed with Seurat (version 3.1.5) as shown in Figs. 3a, 5a, 6b, and Additional file 6: Figure S4b. Initially, we removed the cells with mapped reads of < 2000 and subsequently used read count matrices to conduct the clustering analysis (Fig. 7a). The processed log2CPM matrix was used for the clustering analysis, as depicted in Additional file 6: Figure S4a. We employed a uniform parameter set to perform cluster analyses. The code is listed in Additional file 2.

### Analysis of differentially expressed genes

As shown in Additional file 1: Tables S5, S6, and S8, the "FindMarkers" function of Seurat (version 3.1.5) was employed to detect DEGs between cells. All the parameters were set as defaults, and genes with Bonferroni-corrected *P* values less than 0.05 were selected as DEGs.

### GO term and KEGG pathway enrichment analysis

GO term enrichment analyses on gene sets were conducted (Fig. 5c; Additional file 1: Tables S4, S7, and S9) using DAVID 6.8 as the analysis tool [15]. GO terms from the biological process branch (GOTERM_BP_DIRECT) with Bonferroni-corrected *P* values < 0.05 were selected.

As depicted in Fig. 6d, KEGG pathway enrichment analysis of gene sets was conducted using DAVID 6.8 [15]. Pathways with Bonferroni-corrected *P* values < 0.05 were selected.

## Results

### Identification of gene signatures for proliferative cells and hepatocytes from scRNA-Seq data

The Tabula Muris project has facilitated the sorting of 50,000 cells from 20 organs and tissues of *Mus musculus* using a fluorescence-activated cell sorter (FACS) and provides a compendium of single-cell transcriptomes for 81 *Mus musculus* cell types (Additional file 1: Table S1). The gene expression profiles for each cell type (see "Gene expression data preprocessing" in the "Methods" section) were inferred and used as profiles for their respective human counterparts. In our previous study, we identified 87 genes specifically expressed in stem/progenitor cell types that were associated with cell proliferation functions. These genes were used as a cell-type-specific gene signature for proliferative cells, designated as the "S1 gene signature" (Additional file 1: Table S2). Next, genes expressed in stem/progenitor cell types were excluded, and the remaining genes were clustered (see "Expression heatmap and hierarchical clustering analysis" in the "Methods" section). Consequently, we identified a set of 225 genes showing a specific transcription pattern in hepatocytes (Fig. 1a) and explored their expression patterns across 54 human tissues from the GTEx project [16]. In total, 142 genes (Additional file 1: Table S3), grouped as the "Hep gene signature", showed specific transcription in liver tissue (see "Expression heatmap and hierarchical clustering analysis" and "Gene set expression analysis" in the "Methods" section; Fig. 1b, c). We further investigated the expression of the Hep gene signature, designated "Hep index", in tumor and para-carcinoma tissues of 32 tumor types from The Cancer Genome Atlas (TCGA) project [17]. The Hep gene signature was consistently expressed in tumor and para-carcinoma tissues of HCC (see "Expression heatmap and hierarchical clustering analysis" and "Gene set expression analysis" in the "Methods" section; Fig. 1d, e) and enriched with genes participating in hepatocyte-specific functions (see "GO term and KEGG pathway enrichment analysis" in the "Methods" section; Additional file 1: Table S4). Based on the collective results, the Hep gene signature was inferred as a cell-type-specific gene signature for hepatocytes.

### The hep and S1 gene signatures are respectively associated with the differentiation and proliferation states of hepatocytes

The histological grades of HCC samples in the TCGA project are recorded in the TCGA Clinical Explorer [18]. We categorized the HCC samples into high-, intermediate- or low- tumor-grade groups and calculated the Hep index and S1 index (expression of the S1 gene signature) for samples from each group (see "Gene set expression analysis" in the "Methods" section). Taking *P* < 0.05, we found that the low-tumor-grade group exhibited the highest Hep index, with the second-highest Hep index for the intermediate-tumor-grade group, and the third-highest for the high-tumor-grade group (Fig. 2a). Additionally, the opposite tendency was observed for the S1 index of the three tumor-grade groups. Expression of genes from the Hep and S1 gene signatures tended to be mutually exclusive in HCC tissues from the TCGA project (see "Expression heatmap and hierarchical clustering analysis" in the "Methods" section; Fig. 2b). The Pearson correlation coefficient between the Hep index and S1 index for the samples is − 0.56 (*P* < 0.0001), which indicates that the two indexes were negatively correlated (see "Gene set expression analysis" in the "Methods" section; Fig. 2b).

Gong et al. [19] profiled the gene expression of mouse liver tissues collected from embryonic day 12.5 to postnatal week 8. Expression of genes from the Hep and S1 gene signatures at different time points is illustrated in Fig. 2c (see "Expression heatmap and hierarchical clustering analysis" in the "Methods" section; Fig. 2c). The Pearson correlation coefficient between the Hep index and S1 index is − 0.92 (*P* < 1.29e−06), which confirmed a negative correlation between the Hep and S1 indexes (Fig. 2d). The proliferative ability of liver progenitor cells is downregulated and the functional capacity upregulated during the development process, with a gradual progression into differentiated hepatocytes [20]. The collective findings from HCC and the developing liver support the utility of the Hep and S1 indexes as respective indicators of cell differentiation and the proliferation states of hepatocytes.

### Detection of proliferative hepatocytes from HCC and para-carcinoma tissues

Lu and co-workers conducted 10X genomics scRNA-Seq on tumor and para-carcinoma tissues obtained from ten HCC patients (GSE149614). In our study, cells were selected from para-carcinoma tissue, clustered with the Seurat program [21], and the Uniform Manifold Approximation and Projection (UMAP) technique was employed for visualization (see "Single-cell clustering" in the "Methods" section; Fig. 3a). Various cell-type markers were used to clarify the clusters, including *ALB* for hepatocytes; *PTPRC* for immune cells; *CD79A* for B cells; *EPCAM* for hepatic progenitors; *DCN* for hepatic stellate cells, fibroblasts, and myofibroblasts; and *PTPRB* for hepatic sinusoidal endothelial cells. Integrated marker analysis led to the classification of clusters 6, 14, and 17 as hepatocytes (Fig. 3b).

Next, we calculated the Hep index for individual cells from HCC and para-carcinoma tissues (see "Gene set expression analysis" in the "Methods" section). Cells from para-carcinoma tissue samples were selected, and the Hep index of cells was able to fit a mixture of exponential and normal distributions (Fig. 3c). This suggests that the cells are from different populations, and the cells under normal distribution with a higher Hep index are hepatocytes, and those under exponential distribution are non-hepatocytes. We found that 0.5 is a likely threshold to distinguish the two cell populations, and used it for the identification of hepatocytes. The identified hepatocytes shared the same group of cells in clusters 6, 14, and 17 of the UMAP plot (Fig. 3d).

The S1 index for cells from HCC and para-carcinoma tissues was calculated (see "Gene set expression analysis" in the "Methods" section). Cells with a Hep index > 0.5 were selected as hepatocytes from para-carcinoma tissues. The S1 index of the cells was able to fit an exponential distribution (Additional file 3: Figure S1). Because cells with S1 index > 0.1 were very rare (1%), we arbitrarily selected 0.1 as the threshold for cells with high proliferative ability.

The Hep and S1 indexes were plotted for cells in HCC and para-carcinoma tissues of each patient (see "Gene set expression analysis" in the "Methods" section; Fig. 4). Using 0.5 as the Hep index threshold and 0.1 as the S1 index threshold, we identified proliferative hepatocytes in all HCC tissues, except sample HCC08T. Eight proliferative hepatocytes were detected in the para-carcinoma tissue sample HCC03N.

### Differential gene expression between proliferative hepatocytes from HCC and para-carcinoma tissue samples

We focused on the molecular differences between proliferative hepatocytes from HCC and para-carcinoma tissues, which may elucidate the mechanisms underlying aberrant proliferative signaling in malignant cells. Cells from HCC and para-carcinoma tissues were visualized, and proliferative hepatocytes were identified (see "Single-cell clustering" in the "Methods" section; Fig. 5a, b). Proliferative hepatocytes from five HCC tissue samples (HCC10L, HCC09T, HCC02T, HCC03T, and HCC04T) were located in individual clusters. Additionally, those from three other HCC tissue samples (HCC06T, HCC05T, and HCC01T) shared one cluster, and those from the para-carcinoma tissue sample HCC03N were in an individual cluster (cluster 22).

Differentially expressed genes (DEGs) between proliferative hepatocytes from para-carcinoma and HCC tissues were examined. Overall, 40 genes were upregulated in proliferative hepatocytes from para-carcinoma tissue and none in proliferative hepatocytes from HCC tissue (see "Analysis of differentially expressed gene" in the "Methods" section; Additional file 1: Table S5). GO term enrichment analysis revealed they were enriched in terms related to cellular response to ion and acute phase response (see "GO term and KEGG pathway enrichment analysis" in the "Methods" section; Fig. 5c). We further explored expression patterns of the genes across 54 human tissues from the GTEx project. Notably, 12 of the genes were specifically expressed in liver tissues (Additional file 4: Figure S2). Hepatocytes (normal or malignant) from HCC and para-carcinoma tissues were identified as shown in Fig. 5e. The observed specific expression of these 12 genes in hepatocytes supports their participation in cell-type-specific roles in proliferative hepatocytes.

We subsequently examined the functions of the 12 genes in HCC based on reports in the PubMed database. Three genes downregulated in relation to induction of cell proliferation were identified, specifically, *HAMP* [12], *LINC01093* [22], and *CFHR3* [23]. An earlier study by Ramakrishnan et al. [14] reported that *HAMP*-*SLC40A1* signaling modulates the proliferation of human pulmonary artery smooth muscle cells (hPAMSC). We discovered that *HAMP* was specifically expressed in hepatocytes and *SLC40A1* was specifically expressed in macrophages in HCC and para-carcinoma tissues (Figs. 5e, f, 6a, b). We focused on *HAMP*-*SLC40A1* signaling and intended to clarify the interactions between different cell types and determine their impact on aberrant proliferative signaling in the HCC microenvironment.

*HAMP* was expressed in seven of the eight proliferative hepatocytes from para-carcinoma tissue and three of the 545 proliferative hepatocytes from HCC tissue samples (Fig. 5d). We further investigated *HAMP* expression in all hepatocytes from HCC and para-carcinoma tissues. The previous analysis of the Hep index for cells from para-carcinoma tissue suggested that 0.5 could be an adequate threshold to distinguish hepatocytes from non-hepatocytes (Fig. 3c). Thus, hepatocytes were identified as cells with Hep index > 0.5. Specific *HAMP* expression in hepatocytes from para-carcinoma tissue was confirmed (Fig. 5e, f). DEGs between hepatocytes from para-carcinoma and HCC tissues were detected (see "Analysis of differentially expressed genes" in the "Methods" section; Additional file 1: Table S6). We further conducted GO term enrichment analysis on the 282 upregulated genes in para-carcinoma tissue. The genes were enriched in terms related to cellular response to ion and acute phase response (see "GO term and KEGG pathway enrichment analysis" in the "Methods" section; Additional file 1: Table S7).

### A subset of macrophages expressing *SLC40A1* is specifically present in para-carcinoma tissue

We identified clusters of macrophages in the UMAP plot with the cell-type markers *ITGAM*, *CD68*, *CD163*, *CD14*, and *FCGR3A* (Fig. 6a, b) and observed specific expression of *SLC40A1* in macrophages. Next, macrophages were classified according to their origin (HCC or para-carcinoma tissue), and *SLC40A1* expression status (whether they expressed *SLC40A1* or not) into four groups: (1) from para-carcinoma tissue and expressing *SLC40A1* (N_exp group), (2) from para-carcinoma tissue but not expressing *SLC40A1* (N_noexp group), (3) from HCC tissue and expressing *SLC40A1* (T_exp group), and (4) from HCC tissue but not expressing *SLC40A1* (T_noexp group).

For identified macrophages in the UMAP plot, we counted cell numbers from the four groups (Fig. 6c). In HCC tissue, macrophages from the T_exp and T_noexp groups were located mainly in the same cluster (cluster 3), suggesting that the expression of *SLC40A1* does not influence the transcriptional program of the majority of macrophages. However, in para-carcinoma tissue, most macrophages from the N_exp and N_noexp groups were assembled into distinct cell clusters (Fig. 6c), implying an effect of *SLC40A1* transcription on the gene expression patterns of macrophages. Cluster 10 contained the highest number of *SLC40A1*-expressing macrophages in para-carcinoma tissue (indicated by the column of "N_exp" in Fig. 6c). *SLC40A1*-expressing macrophages were also the predominant cell type in this cluster (indicated by the row of "Cluster10" in Fig. 6c). *HAMP*-*SLC40A1* signaling was only potentially activated in macrophages from the N_exp group because both *HAMP* and *SLC40A1* were expressed under this circumstance. Cluster 10 may therefore present the subset of macrophages linked to *HAMP*-*SLC40A1* signaling.

We further identified marker genes of the cluster by comparison with other macrophages in the UMAP plot (see "Analysis of differentially expressed genes" in the "Methods" section; Additional file 1: Table S8) and conducted GO term as well as KEGG pathway enrichment analyses (see "GO term and KEGG pathway enrichment analysis" in the "Methods" section). Genes implicated in response to various infections were enriched (Fig. 6d, Additional file 1: Table S9), suggesting roles of cluster 10 macrophages in the antimicrobial immune response in the liver. Next, we prioritized marker genes and generated heatmaps of the top 9 markers (*CD5L*, *CETP*, *MARCO*, *CXCL12*, *PLAC8*, *VACM1*, *CFP*, *LYVE1*, and *SDC3*, Fig. 6e). The top genes specifically expressed in macrophages and highly expressed in cluster 10 (*CD5L*, *CETP*, *MARCO*, and *CFP*) were ultimately selected. *CD5L* encodes a secreted protein that is mainly expressed by macrophages in lymphoid and inflamed tissues and regulates the mechanisms underlying inflammatory responses, such as those involved with infection or atherosclerosis [24]. *CETP* is involved in the transfer of neutral lipids, including cholesteryl ester and triglyceride, among lipoprotein particles [25]. *MARCO* encodes a protein that belongs to the class A scavenger receptor family and is part of the innate antimicrobial immune system [26]. It has been proposed that the protein binds Gram-negative and Gram-positive bacteria via an extracellular C-terminal scavenger receptor cysteine-rich (SRCR) domain. *CFP* encodes a plasma glycoprotein that positively regulates the alternative complement pathway of the innate immune system [27]. This protein binds several microbial surfaces and apoptotic cells and stabilizes C3 and C5 convertase enzyme complexes in a feedback loop that ultimately leads to the formation of a membrane attack complex and lysis of target cells. Both *CD5L* and *CETP* participate in lipid metabolism and are related to inflammatory responses, whereas *MARCO* and *CFP* are involved in the antimicrobial immune response. We employed the above four genes as biomarkers of cluster 10 macrophages.

*Emr1* was used as a marker gene of macrophages in mice. We collected *Emr1*-expressing cells from 20 mouse organs and tissues in the Tabula Muris project as macrophages. Cells were clustered with Seurat, the UMAP technique employed for visualization, and those expressing *Marco*, *Cd5l*, and *Cfp* were highlighted (see "Single-cell clustering" in the "Methods" section; Fig. 7a, b). The gene homolog of *CETP* was not identified in mice. Additionally, cluster 15 was the only cell cluster expressing all three marker genes. The liver was determined as the tissue origin of cells in the cluster from the Tabula Muris project. Thus, the subset of macrophages expressing *CD5L*, *CETP*, *MARCO*, and *CFP* appears to be liver-specific, indicating a unique role of *HAMP* in the regulation of macrophages associated with liver function.

### Interactions between *HAMP* from hepatocytes and *SLC40A1* from macrophages are disrupted in HCC

We analyzed *HAMP* and *SLC40A1* expression patterns in two additional scRNA-Seq datasets. Previously, MacParland and co-workers [28] sequenced parenchymal and non-parenchymal cells obtained from fractionation of fresh hepatic tissue from five human livers (GSE115469). We designated this dataset as the "normal liver dataset". Ma et al. [29] sequenced cells from liver cancer biospecimens obtained from nine HCC and ten intrahepatic cholangiocarcinoma patients (GSE125449). This dataset was designated as the "HCC dataset".

We calculated the S1 and Hep indexes for individual cells in the two datasets (see "Gene set expression analysis" in the "Methods" section). The Hep index of cells fits a mixture of exponential and normal distributions in each dataset (Additional file 5: Figure S3a, b). The cells under normal distribution with a higher Hep index are inferred as hepatocytes, and the cells under exponential distribution are non-hepatocytes. We used zero as the threshold in the normal liver dataset and one as the threshold in the HCC dataset to identify hepatocytes. The Hep and S1 indexes of cells in the two datasets were plotted, and cells expressing *HAMP* are highlighted in Additional file 5: Figure S3c and d. Our results showed that 2212 of 3507 hepatocytes in the normal liver dataset and 10 of 320 hepatocytes in the HCC dataset expressed *HAMP*. Furthermore, with the S1 index > 0.1 as a threshold for proliferative cells, we found that 14.5% of proliferative cells in the normal liver dataset and 1.7% of proliferative cells in the HCC dataset expressed *HAMP*. The significant differences in *HAMP*-expressing cells between the two datasets reflect the on/off state of *HAMP* expression in normal liver and HCC tissue.

Cells in each dataset were clustered with Seurat, and the UMAP technique was employed for visualization (see "Single-cell clustering" in the "Methods" section; Additional file 6: Figure S4a, b). We highlighted cells with a Hep index above the threshold and identified the clusters belonging to hepatocytes. Macrophages with *ITGAM*, *CD68*, *CD163*, *CD14*, and *FCGR3A* expression were identified, and cells expressing *CD68* are highlighted in the UMAP plots. *HAMP* was specifically expressed in hepatocytes from normal liver tissue. For *SLC40A1*-expressing macrophages in each dataset, we calculated the percentage of cells expressing *CD5L*, *CETP*, *MARCO*, and *CFP*. Our data showed that > 35% of macrophages in the normal liver dataset but < 16% of macrophages in the HCC dataset expressed *CETP*, *MARCO*, and *CFP* (Additional file 1: Table S10). In Lu and co-workers’ scRNA-Seq dataset (GSE149614), > 66% of macrophages in para-carcinoma tissue expressed *CD5L*, *CETP*, *MARCO*, and *CFP*, in contrast to < 22% of macrophages in HCC tissue.

UMAP plots of the three scRNA-Seq datasets suggest that *HAMP* is specifically expressed in hepatocytes and *CD5L*, *CETP*, *MARCO*, and *CFP* are predominantly expressed in macrophages (Figs. 5e, f, 6b, e, Additional file 6: Figure S4a, b). We employed two large-scale bulk HCC RNA-Seq/microarray datasets to compare the expression of *HAMP*, *CD5L*, *CETP*, *MARCO*, and *CFP* between HCC and para-carcinoma tissue samples. One dataset is from the TCGA project containing 369 HCC and 50 para-carcinoma tissue samples and the other is from the study of Lim et al. [30] containing 240 HCC and 193 para-carcinoma tissue samples (GSE36376). Notably, all five genes were significantly downregulated in HCC tissues (Additional file 7: Figure S5).

## Discussion

Based on previous and current findings, we speculate that in the HCC environment, downregulation of *HAMP* in hepatocytes activates the iron export channel *SLC40A1* on macrophages, which subsequently promotes iron transport from macrophages and fuels cancer cells with iron to sustain their proliferative ability. Moreover, *HAMP*-*SLC40A1* signaling may induce a subset of macrophages to initiate responses to various infections. The downregulation of *HAMP* may lead to the disappearance of this subset of macrophages and consequent weakening of antimicrobial activity in the HCC microenvironment. Although our current experiments elucidate the involvement of *HAMP*-*SLC40A1* signaling in the HCC microenvironment, further wet laboratory experiments are warranted to sort the subsets of macrophages responding to changes in *HAMP* expression and clarify their functions.

In addition to *HAMP*, it has also been reported that *TFR1* (*TFRC*), *TFR2*, *HFE*, *HJV* (*HFE2*), and *SLC40A1* play important roles in the iron metabolism process [31]. Evaluation of the expression of *TFR1* (*TFRC*), *TFR2*, *HFE*, *HJV* (*HFE2*), and *SLC40A1* in proliferative hepatocytes of HCC and para-carcinoma tissues (Additional file 8: Figure S6) revealed no significant differences in expression between the two types of proliferative hepatocytes and between hepatocytes from HCC and para-carcinoma tissues (Additional file 1: Tables S5, S6). These data suggest that the iron metabolism ability associated with these genes is not different between hepatocytes from HCC and para-carcinoma tissues. Previous studies have reported that *HAMP* limits iron flux to the bloodstream by promoting degradation of the iron exporter *SLC40A1* in target cells [32]. The lack of *HAMP* may contribute to increased iron flux from circulating macrophage cells in the HCC microenvironment. Whereas the iron metabolism ability of proliferative hepatocytes from tumor and para-carcinoma tissues may be similar, the iron flux around the cells may differ; although confirmatory evidence supporting this theory is lacking.

Here, we used an *in silico* approach to identify proliferative cells in tumor and para-carcinoma tissues from scRNA-Seq data. Determination of the molecular differences between the two proliferative cell types aids in clarifying the mechanisms underlying aberrant proliferative signaling and provides druggable targets, which may be of significant interest to researchers focused on evaluating tumors in silico with scRNA-Seq.

## Conclusion

The *HAMP*-*SLC40A1* signaling between hepatocytes and macrophages is disrupted in the HCC microenvironment, which contributes to the aberrant proliferation of hepatocytes. However, these conclusions are from in silico analysis and require further validation with wet laboratory experiments.

## Supplementary Information

**Additional file 1.** Tables S1–11.

**Additional file 2.** The R code to conduct single-cell clustering analysis.

**Additional file 3.** **Figure S1**: Distribution of the S1 index of hepatocytes identified in para-carcinoma tissue.

**Additional file 4.** **Figure S2**: The 40 upregulated genes in 54 human tissue samples from the GTEx project.

**Additional file 5.** **Figure S3**: Identification of hepatocytes based on the Hep index in normal liver and HCC datasets. **a.** Distribution of the Hep index of all cells in the normal liver dataset. **b.** Distribution of the Hep index of all cells in the HCC dataset. **c.** Scatter plot of S1 and Hep indexes for cells in the normal liver dataset. Each dot represents a cell. The cells expressing *HAMP* are highlighted. **d.** Scatter plot of S1 and Hep indexes for cells in the HCC dataset. Each dot represents a cell. The cells expressing *HAMP* are highlighted.

**Additional file 6.** **Figure S4**: Heatmaps showing expression of *HAMP*, *CD5L*, *CETP*, *MARCO*, and *CFP* in cells from normal liver and HCC datasets. **a.** Expression of *HAMP*, *CD5L*, *CETP*, *MARCO*, and *CFP* in the normal liver dataset. Cells with a Hep index above the threshold are highlighted to indicate clusters belonging to hepatocytes in the UMAP plot, along with cells expressing *CD68* and *SLC40A1* to indicate *SLC40A1*-expressing macrophages. **b.** Expression of *HAMP*, *CD5L*, *CETP*, *MARCO*, and *CFP* in the HCC dataset.

**Additional file 7.** **Figure S5**: Expression of *HAMP*, *MARCO*, *CETP*, *CD5L*, and *CFP* in para-carcinoma and HCC tissue samples. **a.** Expression of *HAMP*, *MACRO*, *CD5L*, *CETP*, and *CFP* in para-carcinoma and HCC tissue samples from the HCC dataset of the TCGA project. **b.** Expression of *HAMP*, *MACRO*, *CD5L*, *CETP*, and *CFP* in para-carcinoma and HCC tissue samples from the HCC dataset GSE36376. Two independent probe sets were designed for the *CETP* gene. N and T denote samples from para-carcinoma and HCC tissue, respectively. The *t*-test was applied to compare gene expression between two sample types. *** Indicates P values < 0.001.

**Additional file 8.** **Figure S6**: Expression of *TFR1* (*TFRC*), *TFR2*, *HFE*, *HJV* (*HFE2*), and *SLC40A1* in proliferative hepatocytes from para-carcinoma and HCC tissue samples. Proliferative hepatocytes from para-carcinoma tissue are indicated in the UMAP plot. The other cells are proliferative hepatocytes from HCC tissue.

## Data Availability

All the data are publicly available. The human scRNA-Seq data, mouse liver development microarray data, and human HCC microarray data are available in the GEO repository [https://www.ncbi.nlm.nih.gov/geo], with accession numbers GSE149614, GSE115469, GSE125449, GSE132034, and GSE36376. The Tabula Muris mouse scRNA-Seq data are available at GitHub [https://github.com/czbiohub] under the account of Chan Zuckerberg Biohub with URL [https://github.com/czbiohub/tabula-muris-vignettes/tree/master/data]. The TCGA human tumor and para-carcinoma tissue RNA-Seq data are available at the FIREHOSE database [http://gdac.broadinstitute.org/]. The TCGA human tumor tissue histology grade data are available at The Cancer Genome Atlas Clinical Explorer [http://genomeportal.stanford.edu/pan-tcga/]. The GTEx human tissue RNA-Seq data are available at the GTEx database [https://gtexportal.org/home/datasets]. The details regarding the data, the name, and path to the downloaded files are listed in Additional file 2: Table S11.

## Abbreviations

HCC: Hepatocellular carcinoma
scRNA-Seq: Single-cell RNA sequencing
MCA: Mouse Cell Atlas
GTEx: Genotype-tissue expression
FACS: Fluorescence-activated cell sorter
TCGA: The Cancer Genome Atlas
UMAP: Uniform manifold approximation and projection
DEGs: Differentially expressed genes
hPAMSC: Human pulmonary artery smooth muscle cell
RP10K: Reads per 10 kilo
CPM: Counts per million
TPM: Transcripts per million
FPKM: Fragments per kilobase of transcript per million mapped reads
