# Supplementary figures and images for: In silico analysis suggests disruption of interactions between HAMP from hepatocytes and SLC40A1 from macrophages in hepatocellular carcinoma
Source: BMC Med Genomics. 2021 May 17;14:128. doi: 10.1186/s12920-021-00977-0 (PMC8130390; doi:10.1186/s12920-021-00977-0)

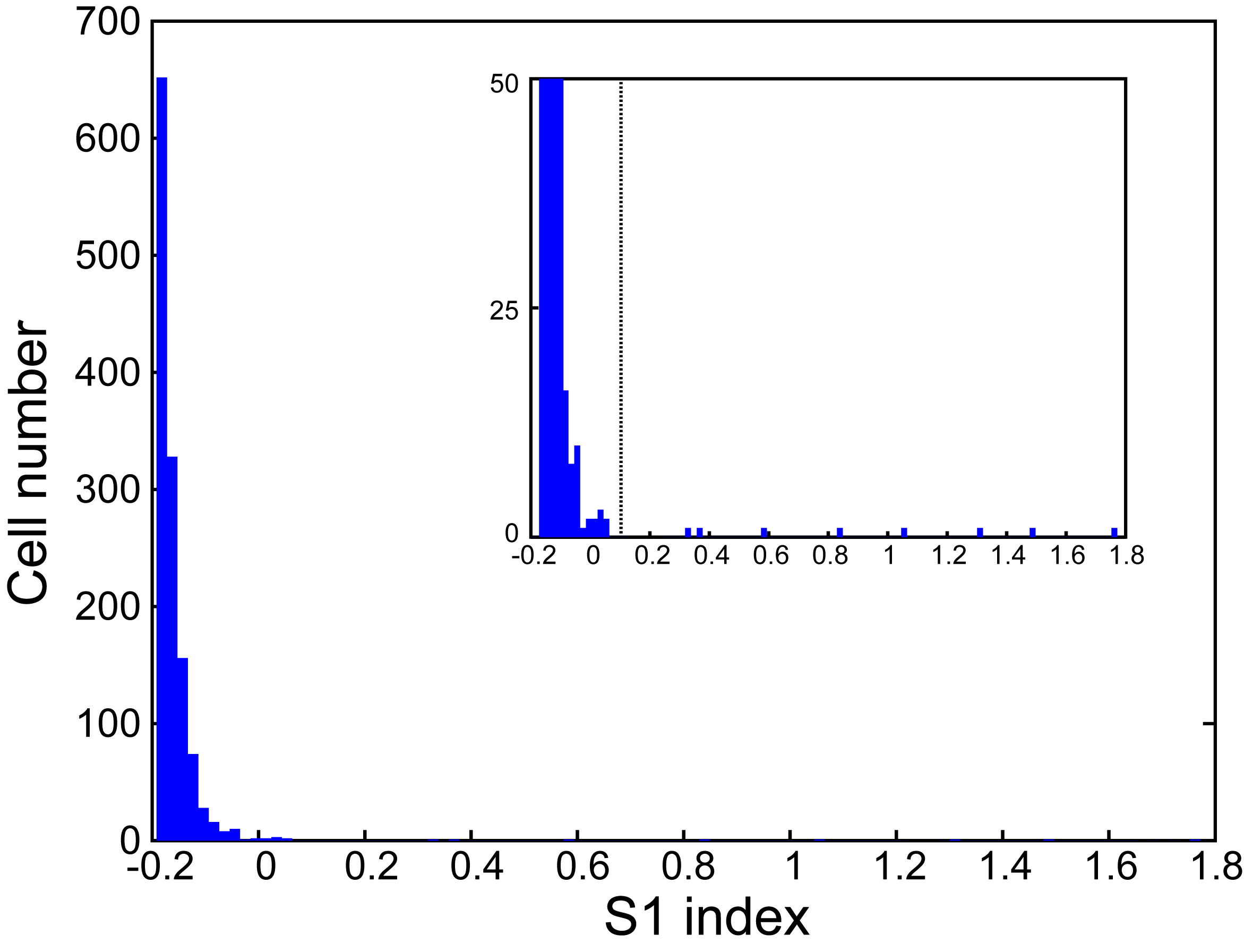

Supplement: Supplementary file 3 — Additional file 3. Figure S1: Distribution of the S1 index of hepatocytes identified in para-carcinoma tissue. [file 12920_2021_977_MOESM3_ESM.jpg]

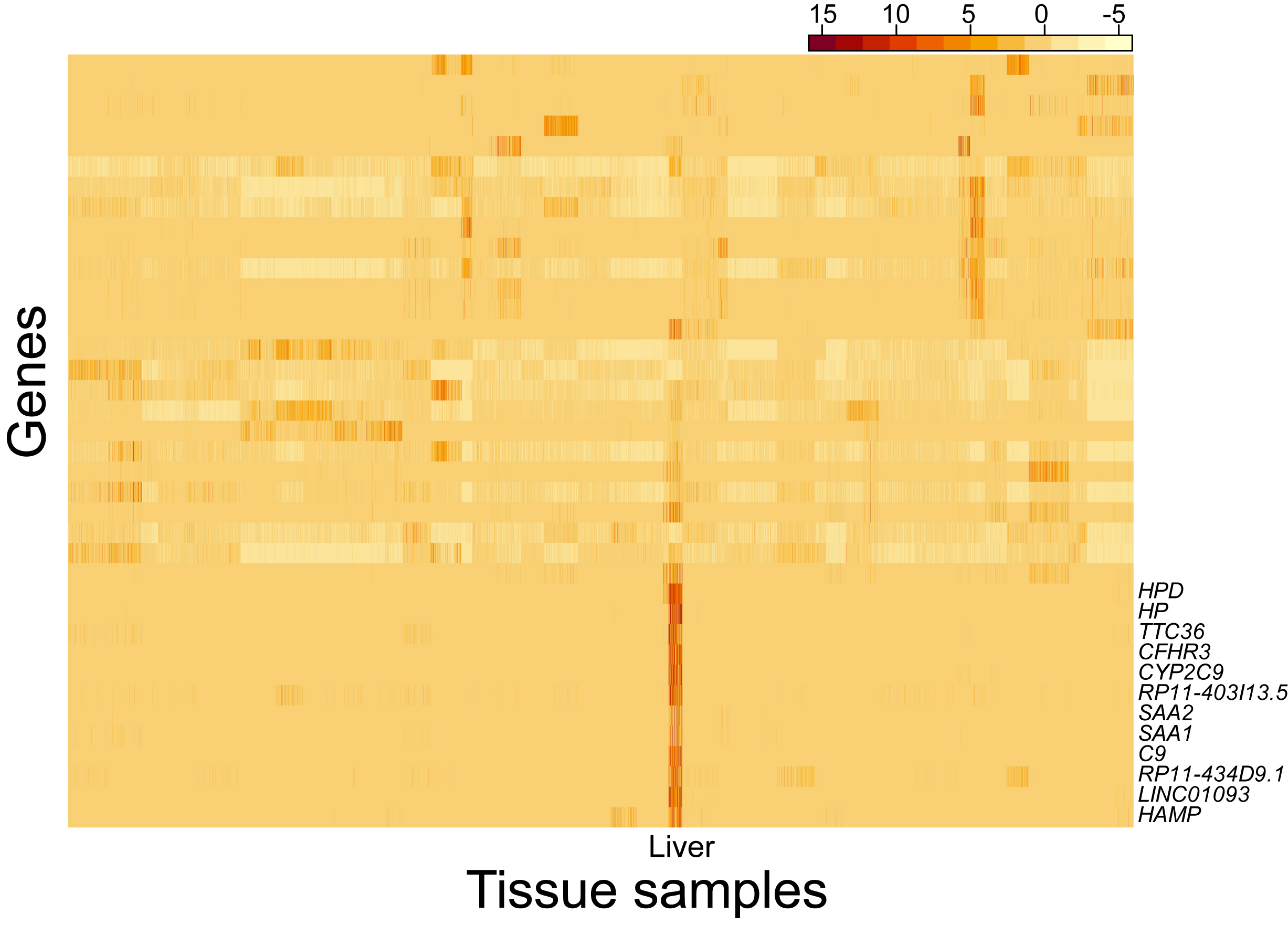

Supplement: Supplementary file 4 — Additional file 4. Figure S2: The 40 upregulated genes in 54 human tissue samples from the GTEx project. [file 12920_2021_977_MOESM4_ESM.jpg]

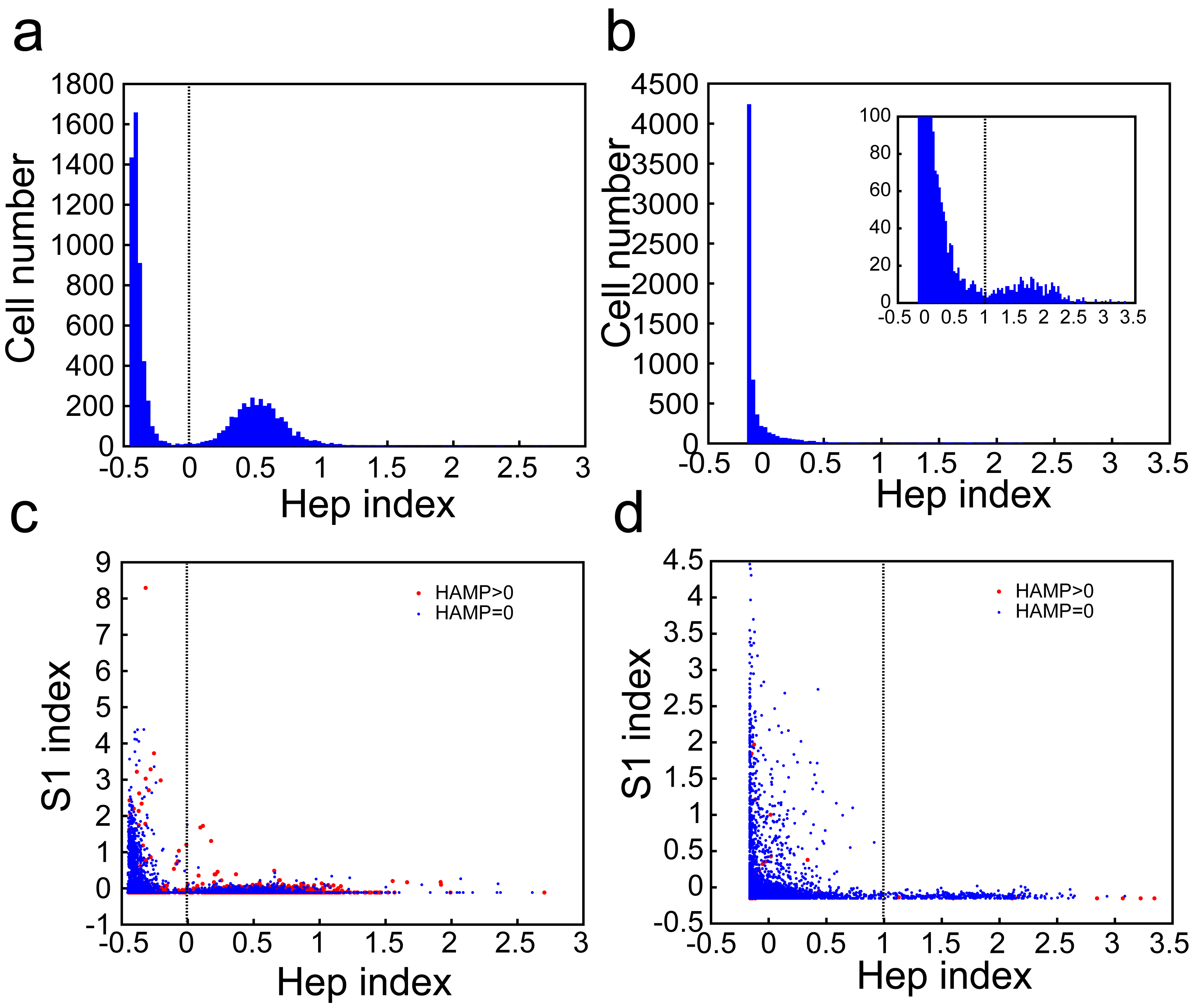

Supplement: Supplementary file 5 — Additional file 5. Figure S3: Identification of hepatocytes based on the Hep index in normal liver and HCC datasets. a. Distribution of the Hep index of all cells in the normal liver dataset. b. Distribution of the Hep index of all cells in the HCC dataset. c. Scatter plot of S1 and Hep indexes for cells in the normal liver dataset. Each dot represents a cell. The cells expressing HAMP are highlighted. d. Scatter plot of S1 and Hep indexes for cells in the HCC dataset. Each dot represents a cell. The cells expressing HAMP are highlighted. [file 12920_2021_977_MOESM5_ESM.jpg]

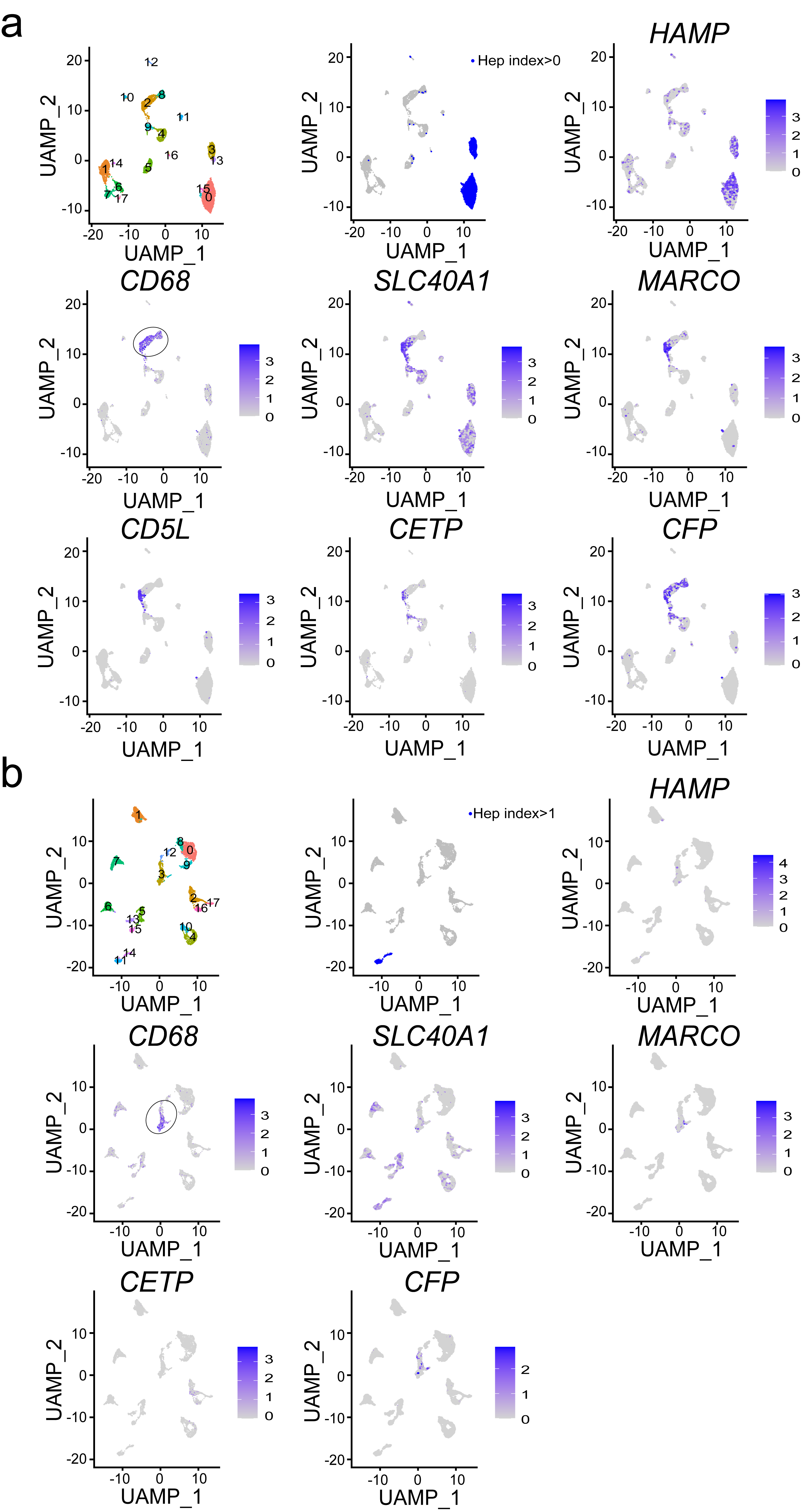

Supplement: Supplementary file 6 — Additional file 6. Figure S4: Heatmaps showing expression of HAMP, CD5L, CETP, MARCO, and CFP in cells from normal liver and HCC datasets. a. Expression of HAMP, CD5L, CETP, MARCO, and CFP in the normal liver dataset. Cells with a Hep index above the threshold are highlighted to indicate clusters belonging to hepatocytes in the UMAP plot, along with cells expressing CD68 and SLC40A1 to indicate SLC40A1-expressing macrophages. b. Expression of HAMP, CD5L, CETP, MARCO, and CFP in the HCC dataset. [file 12920_2021_977_MOESM6_ESM.jpg]

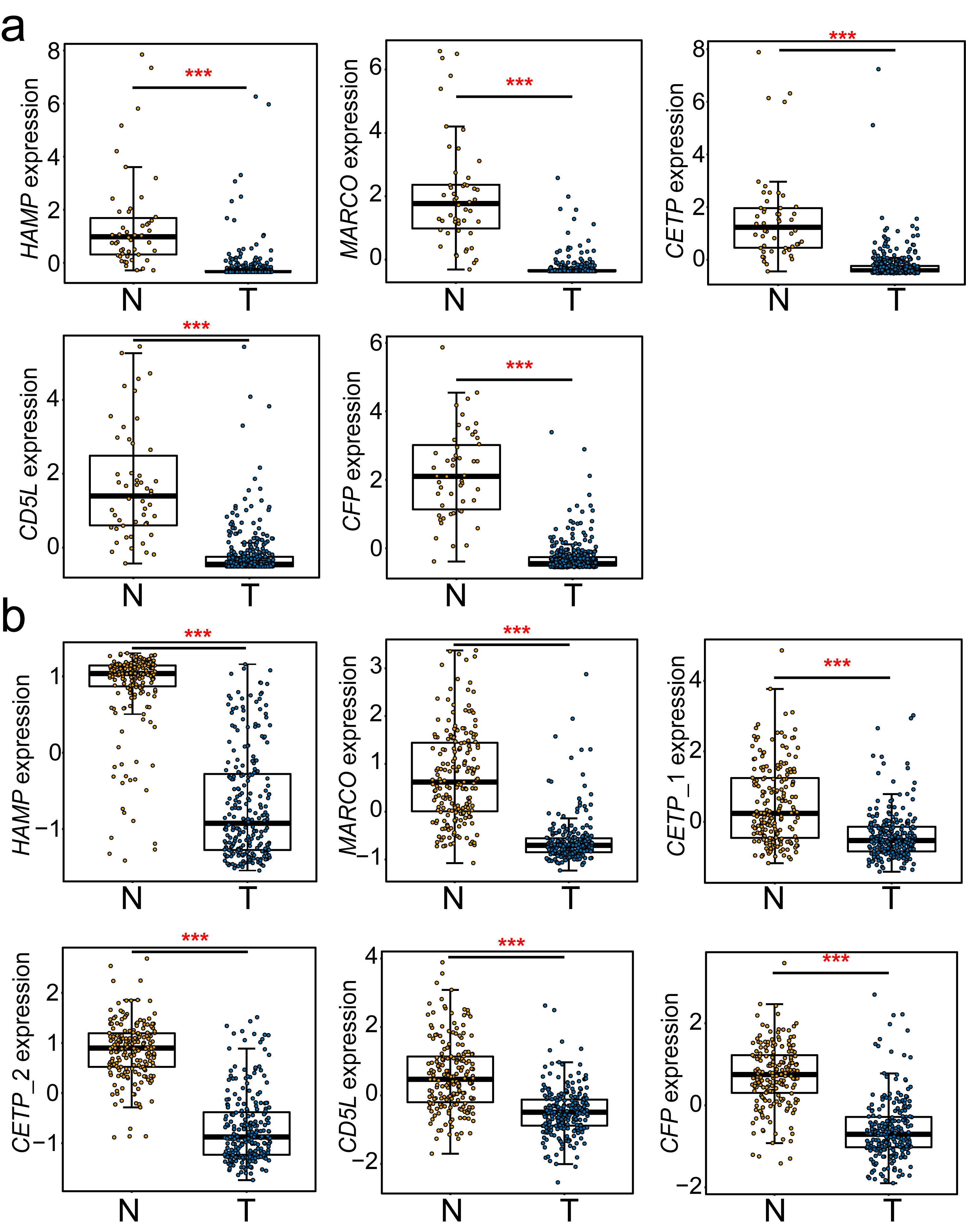

Supplement: Supplementary file 7 — Additional file 7. Figure S5: Expression of HAMP, MARCO, CETP, CD5L, and CFP in para-carcinoma and HCC tissue samples. a. Expression of HAMP, MACRO, CD5L, CETP, and CFP in para-carcinoma and HCC tissue samples from the HCC dataset of the TCGA project. b. Expression of HAMP, MACRO, CD5L, CETP, and CFP in para-carcinoma and HCC tissue samples from the HCC dataset GSE36376. Two independent probe sets were designed for the CETP gene. N and T denote samples from para-carcinoma and HCC tissue, respectively. The t-test was applied to compare gene expression between two sample types. *** Indicates P values < 0.001. [file 12920_2021_977_MOESM7_ESM.jpg]

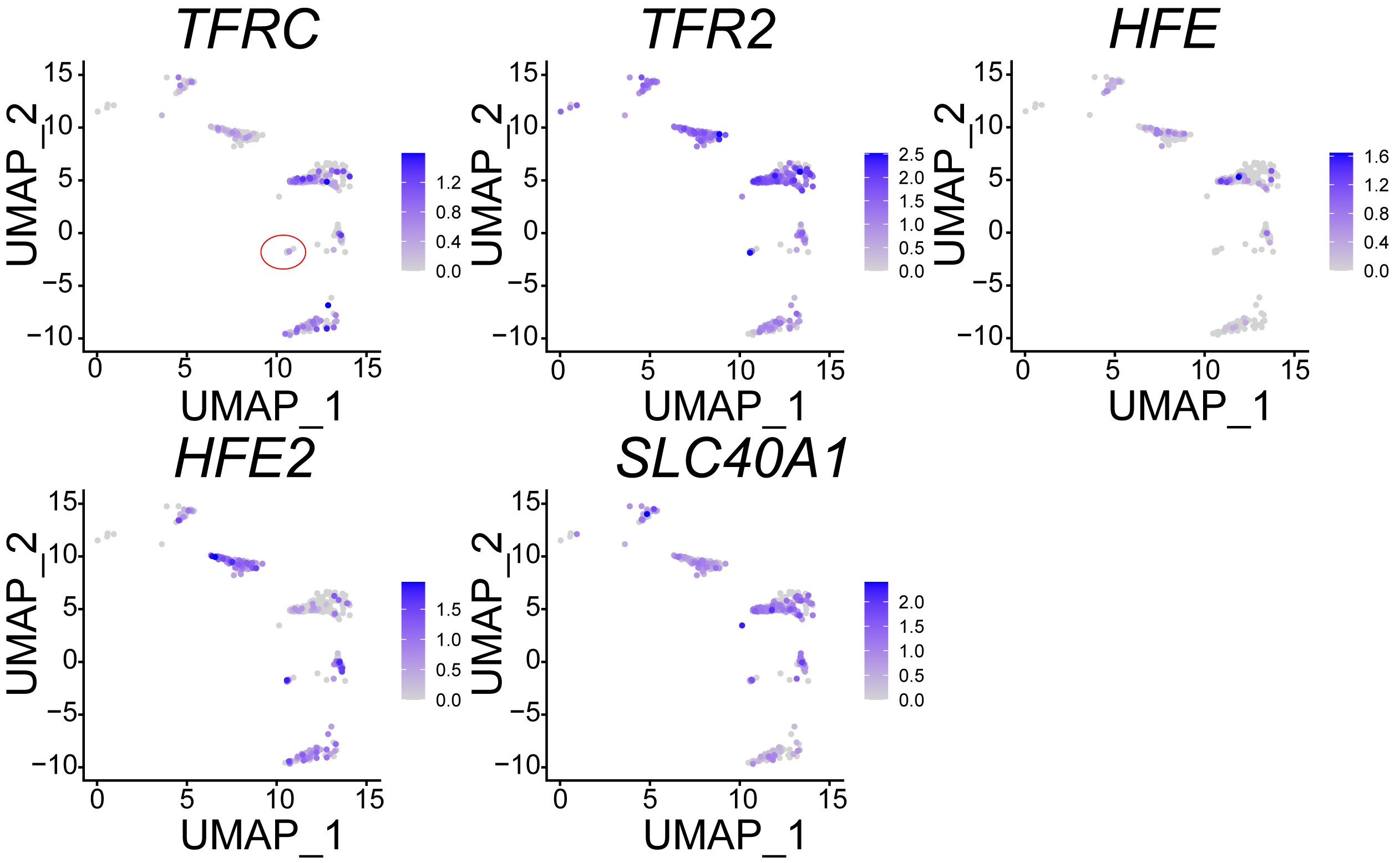

Supplement: Supplementary file 8 — Additional file 8. Figure S6: Expression of TFR1 (TFRC), TFR2, HFE, HJV (HFE2), and SLC40A1 in proliferative hepatocytes from para-carcinoma and HCC tissue samples. Proliferative hepatocytes from para-carcinoma tissue are indicated in the UMAP plot. The other cells are proliferative hepatocytes from HCC tissue. [file 12920_2021_977_MOESM8_ESM.jpg]
